# Editorial: Global excellence in cellular neuropathology: Europe

**DOI:** 10.3389/fncel.2023.1289444

**Published:** 2023-10-25

**Authors:** Ryszard Pluta, Aurel Popa-Wagner, Dirk M. Hermann

**Affiliations:** ^1^Department of Pathophysiology, Medical University of Lublin, Lublin, Poland; ^2^Center of Experimental and Clinical Medicine, University of Medicine and Pharmacy, Craiova, Romania; ^3^Department of Neurology, University Hospital Essen, University of Duisburg-Essen, Essen, Germany

**Keywords:** encephalitis, epilepsy, DRAVET syndrome, stem cell-derived neurons, therapy, new strategies

Cellular neurosciences have their genuine research focuses in different parts of this world. Local research histories, the focus of leading institutes and national funding opportunities shaped research priorities in various countries in diverse ways. After Frontiers in Cellular Neuroscience had organized several Research Topics with a focus on specific research contents, the journal decided to present to the scientific community latest achievements by internationally renowned scientists in Research Topics focusing on defined world regions. The main aim of this initiative was to provide an overview on research landscapes in different parts of the world. Within the Research Topic “*Global excellence in cellular neuropathology: Europe*,” Frontiers in Cellular Neuroscience invited European scientists to present academic excellence, scientific quality, research procedures and state-of-the-art technologies to its readership. This Research Topic was organized by the authors of this editorial, who have their origin in Central Europe (Poland), Eastern Europe (Romania) and Western Europe (Germany), respectively.

To provide representative insights into Cellular Neuropathology, papers were requested to cover major brain disease areas, such as neurodegenerative diseases, ischemic stroke and vascular cognitive disorders, neuroinflammatory and autoimmune brain diseases, brain trauma, brain tumors, epilepsy, age-related neurodegenerative diseases, mood disorders and schizophrenia. Papers were expected to provide knowledge about the neurobiology of diseases, mechanisms of neuronal damage and plasticity, as well as roles of glia in disease processes. This paper Research Topic was intended to shed light on current progress in the field and to reflect future challenges and solutions for cross-border studies. This procedure was an attempt to bring together researchers from different laboratories through this Research Topic. The ultimate goal of this paper Research Topic was to provide an information repository and reference, and to create a platform for the publication of cutting-edge studies that expand our understanding of neurological diseases and disease treatments. The underlying assumption is that advances in molecular, cellular and systems neurosciences will provide prospects for the development of new treatments, which the world is eagerly awaiting. Indeed, the incidence of central nervous system diseases in humans is constantly increasing world-wide. Through the use of genomics, proteomics and high-throughput screening tools, remarkable advances have been made over the past 25 years in disease diagnostics and disease treatment. Although the neuropathology of several diseases including encephalitis, epilepsy and Dravet syndrome is quite well known, until recently we knew little about their molecular mechanisms. Within five original research articles published in this Research Topic, disease mechanisms were evaluated in *in vivo* and *in vitro* studies. Thereby emerging diagnostic methods and therapeutic targets were outlined. In the following, we would like to summarize the contents of these papers.

The publication by Li et al. focused on the functional role of autoantibodies against cerebral blood vessels in patients with GABA_A_ and NMDA receptor-associated encephalitis. Using immunohistochemistry, 149 human IgG monoclonal antibodies from the cerebrospinal fluid of six patients with various forms of autoimmune encephalitis were tested on mouse brain sections for their binding to blood vessels. Antibodies were assessed for their reactivity with purified brain blood vessels by evaluating effects on transendothelial electrical resistance and tight junction protein expression using hCMEC/D3 human brain microvascular endothelial cells as a model of the blood-brain barrier *in vitro*. One vascular-reactive antibody was administered intrathecally to mice to study *in vivo* binding and effects on tight junction proteins such as occludin. Target protein identification was performed using transfected HEK293 cells. Six of the antibodies reacted with blood vessels of the brain, three were from the same patient with GABA_A_ receptor encephalitis, and the remaining three were from different patients with NMDA receptor encephalitis. In case of the monoclonal antibody mAb 001-138, antibody administration to hCMEC/D3 cells resulted in a decrease in transendothelial electrical resistance and a decrease in occludin expression. The functional significance *in vivo* was confirmed in animals treated with mAb 011-138, which induced a decrease in occludin levels. Finally, the unconventional myosin-X was identified as a new autoimmune target of this antibody. This study suggests that autoantibodies against blood vessels may contribute to blood-brain barrier damage, indicating their potential neuropathological relevance.

Dravet syndrome is a rare autosome encephalopathy with epilepsy associated with Nav1.1 channel mutations and defective GABAergic signaling. There is a lack of effective therapies for this syndrome, which requires a better understanding of the mechanisms involved. In a mouse model of Dravet syndrome, Goisis et al. studied GABA tonic currents in brain sections of developing mice before spontaneous seizure onset. In neurons of the temporal cortex and the CA1 area, GABA tonic currents were reduced in mice with Dravet syndrome compared to controls, while in the entorhinal cortex these currents were not affected. However, in this region, the amplification of tonic GABA currents by the neurosteroid and positive allosteric modulator of the GABA_A_ receptor allopregnanonol was reduced in Dravet mice, suggesting altered extrasynaptic GABA_A_ subunits that contributed to this action. Using 4,5,6,7-tetrahydroisoxazolo[5,4-c]pyridin-3-ol (THIP) as a selective GABA agonist, Goisis et al. found decreased δ-subunit-mediated tonic currents in the entorhinal cortex of Dravet syndrome mice. Unexpectedly, in the dentate gyrus, a region of high expression of the δ-subunit, THIP-induced currents in Dravet syndrome mice were greater than in controls. Immunofluorescence study confirmed that δ-subunit expression was decreased in the entorhinal cortex and increased in the dentate gyrus of Dravet mice. Finally, given the importance of neuroinflammation in epilepsy and neurodevelopmental disorders, the authors evaluated classical markers of glial activation. Their data indicate that mice with Dravet syndrome have increased microglia and astrocyte activation in the entorhinal cortex and dentate gyrus compared to controls. In summary, prior to spontaneous seizures, mice with Dravet syndrome develop changes in GABA tonic currents and glial cell activation. Better understanding of the mechanisms involved in these changes during maturation and disease progression may reveal new therapeutic targets.

Selective loss of inhibitory interneurons results in excitatory dominance and may be critical in triggering epileptic activity. Research on mesial temporal lobe epilepsy has so far focused mainly on changes in the hippocampus, including loss of inhibitory interneurons, with less attention paid to the subiculum as the main area of efferents originating in the hippocampus. The subiculum has been shown to occupy a key position in the epileptic network, but data on cellular changes have been controversial. Using a murine intrahippocampal kainate model for mesial temporal lobe epilepsy that exhibits features of human mesial temporal lobe epilepsy such as unilateral hippocampal sclerosis and granule cell dispersion, Franz et al. identified cell loss in the subiculum and quantified changes in specific subpopulations of inhibitory interneurons along the dorsal-ventral axis. They performed an assessments of the hippocampus for degenerated neurons by Fluoro Jade C staining shortly after status epilepticus, fluorescent *in situ* hybridization for glutamic acid decarboxylase 67 mRNA, and immunohistochemistry of neuronal nuclei, parvalbumin, calretinin, and neuropeptide Y on day 21 after kainate model. The authors found a remarkable loss of cells in the ipsilateral subiculum soon after status epilepticus, which was reflected in a reduced density of neuronal cell nuclei in the chronic phase when epileptic activity occurred in the subiculum and simultaneously in the hippocampus. In addition, they showed a reduction in inhibitory interneurons by 50% expressing glutamic acid decarboxylase along the dorsal-ventral axis as well as the transverse subiculum axis. This was especially the case for interneurons that inhibited parvalbumin and, to a lesser extent, calretinin. The density of neuropeptide Y-positive neurons was increased, but double-labeling of glutamic acid decarboxylase mRNA expression revealed that the basis for this was increased or de novo neuropeptide Y expression in non-GABAergic cells with a concomitant reduction of neuropeptide Y-positive inhibitory interneurons. These data suggest a site-specific and cell type-specific response. The susceptibility of basal inhibitory interneurons in mesial temporal lobe epilepsy may contribute to hyperexcitability and epileptic activity.

Human induced pluripotent stem cells represent a promising approach to study the treatment of neurological diseases. Most methods of recording the activity of these cells have serious drawbacks because they are invasive or do not allow the separation of individual cells. Genetically encoded voltage indicators pave the way for high-performance visualization of undisturbed neuronal activity. Conventionally, however, genetically encoded voltage indicators disrupt membrane integrity by inserting multiple copies of transmembrane domains into the cell membrane. To bypass these additions in the cytoplasmic membrane, Alich et al. used a minimally invasive, novel hybrid dark quencher (that is, a genetically encoded voltage indicator) to record the physiological and pathological firing patterns of human-induced pluripotent sensory neurons from patients with congenital erythromelalgia, a chronic pain condition associated with recurrent attacks of redness and swelling in the distal extremities. The authors observed significant differences in the firing patterns of the action potential between the patient's neurons and control neurons. Their system performed well in forebrain neurons derived from human induced pluripotent stem cells, where it detected spontaneous synchronous bursting behavior, thus opening the door to future applications in other cell types and disease models related to impaired neuronal activity and synchronization, e.g., in Alzheimer's disease, Parkinson's disease or epilepsy.

The clinical spectrum of neurodevelopmental disorders associated with variants of the *GRIN* gene results from gene-dependent and variant-dependent changes in the NMDA receptor, interfering with glutamatergic neurotransmission. Although functional annotations of *GRIN* gene variants are critical for stratification and precision/personal medicine design, genetically diagnosed pathogenic GRIN variants outnumber their relative functional annotations. Based on high-resolution 3D crystal models and topological domains conservation between the GluN1, GluN2A and GluN2B subunits of the NMDAR, Santos-Gómez et al. generated a GluN1-GluN2A-GluN2B subunit structural superimposition model to find equivalent positions between GluN subunits. The authors also developed a *GRIN* gene structural algorithm that predicts functional changes in the equivalent structural positions in other GluN subunits. The GRIN structural algorithm was computationally validated against the full repertoire of *GRIN* missense variants, consisting of 4,525 variants. Analysis of this structure-based model revealed absolute predictive power for the GluN1, GluN2A, and GluN2B subunits, both in terms of association of pathogenicity (mild vs. pathogenic variants) and functional impact (loss of function, mild, and gain of function). In addition, they experimentally validated this computational algorithm using an *in silico* library of artificial GluN2B variants equivalent to artificial GluN2A, designed from pathogenic GluN2B variants. Thus, the implementation of the structured GRIN algorithm computationally predicts the pathogenicity and functional annotations of GRIN variants, resulting in duplication of assignments of pathogenic GRIN variants, a 30% reduction in GRIN variants of uncertain significance, and a 70% increase in functionality with annotated GRIN variants. Finally, the GRIN structural algorithm has been implemented in the GRIN Variant Database (http://lmc.uab.es/grindb), providing a computational tool to accelerate the stratification of GRIN missense variants, contributing to clinical decisions regarding the treatment of this neurodevelopmental disorder.

In summary, this Research Topic put together manuscripts on rare neuropathological diseases, for which clinically relevant disease mechanisms have been identified, which had previously been unknown. The insights of these studies will facilitate the proper diagnostics of these diseases, and it will help to develop treatments that alleviate these disease states. Some of the methods used can be adopted for studying other autoimmune or genetic brain diseases. We thank the authors for their contributions and hope that each article will foster further research interest that refines our neuropathological understanding to the benefits of affected patients.

## Author contributions

RP: Writing—original draft. DH: Project administration, Writing—review & editing. AP-W: Writing—review & editing.

